# A Sensitive and Efficient Method for Determination of Capecitabine and Its Five Metabolites in Human Plasma Based on One-Step Liquid-Liquid Extraction

**DOI:** 10.1155/2019/9371790

**Published:** 2019-01-03

**Authors:** Zhipeng Wang, Xinxing Li, Yang Yang, Feng Zhang, Mingming Li, Wei Chen, Shouhong Gao, Wansheng Chen

**Affiliations:** ^1^Department of Pharmacy, Changzheng Hospital, Second Military Medical University, Shanghai 200003, China; ^2^Department of General Surgery, Changzheng Hospital, Second Military Medical University, Shanghai 200003, China

## Abstract

Colorectal cancer is the most common critical disease both in the developed and developing countries. Capecitabine, which has served in clinical practice at least for 10 years, is a first-line antidigestive tract cancer drug for its better efficacy, patient compliance, and lower side effects. An ultra-high-performance liquid chromatography tandem mass spectrometry (UHPLC-MS/MS) method has been developed and completely validated for simultaneous determination of capecitabine and its five metabolites in human plasma from colorectal cancer patients after administration of capecitabine tablet. One-step liquid-liquid extraction was successfully applied using ethyl acetate and isopropanol (19 : 1, V : V) for sample pretreatment. Chromatographic separation was achieved within 5 min based on an Atlantis T3-C_18_ column (3.0 *µ*m, 2.1 × 100 mm) with gradient elution using mobile phases consisting of 0.0075% formic acid in water (pH 4) and in acetonitrile, and the flow rate was 0.3 mL/min. Linear range was approximately 20.0–5000.0 ng/mL for all analytes. Linear correlation coefficients were >0.99 for all regression curves. The intraday and interday accuracy and precision of the method were within ±15.0% and less than 15.0%, respectively. The mean recovery and matrix effect as well as stability of all the analytes ranged from 59.27% to 90.15% and from 74.84% to 114.48% as well as within ±15.0%. This simple, rapid, and sensitive method was successfully applied in 42 sparse clinical samples to verify its practicability.

## 1. Introduction

**Figure 1:**
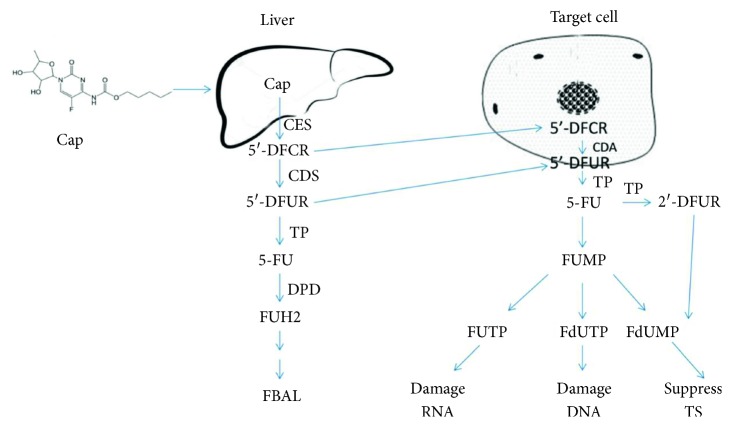
The metabolic pathways and targets of Cap. Cap: capecitabine; 5'-DFCR: 5'-deoxy-5-fluorocytidine; 5'-DFUR: doxifluridine; 2'-DFUR: 5-fluoro-2'-deoxyuridine; 5-FU: 5-fluorouracil; FUH2: dihydrofluorouracil; FBAL: *α*-fluoro-*β*-alanine; CES: carboxylesterase; CDA: cytidine deaminase; TP: thymidylate phosphorylase; TS: thymidylate synthetase; FUMP: fluorouridine monophosphate; FUTP: fluorouridine triphosphate; FdUTP: deoxyfluorouridine triphosphate; FdUMP: deoxyfluorouridine monophosphate.

Colorectal cancer is the leading cause of death in both developed countries and developing countries, and heavy social and economic burden have been brought by this malignant disease. According to the GLOBOCAN2012, colorectal cancer ranked third in new cancer cases worldwide, and second in the developed countries, with estimated 1.4 million cases and 693,900 deaths occurred in 2012 worldwide [1]. In China, there were 310,244 new colorectal cancer cases, which ranked fourth in all new cancer cases (9.20%), and the people who died of colorectal cancer was 149,722 (7.09%), ranking fifth in all cancer death, according to the Chinese Cancer Registry Annual Report. From 1998, the morbidity and mortality of colorectal cancer has been gradually increasing in China [2]. Capecitabine (Cap), which was approved in 2005 by the FDA for the treatment of Dukes' C stage colorectal cancer in adjuvant setting, has turned into the cornerstone for anticolorectal cancer as a prodrug of 5-FU in recent years [3]. As an oral administration prodrug, three metabolic steps were needed to catabolize itself to the active agent 5-FU both in liver and target cells. After administration, Cap was almost totally absorbed in the intestine as an intact molecule and then transferred to the liver by circulatory blood. In liver, Cap was metabolized by carboxylesterase to 5'-deoxy-5-fluorocytidine (5'-DFCR), and then, cytidine deaminase transforms the 5'-DFCR to doxifluridine (5'-DFUR), and this step could also be detected in other normal or tumor tissues. 5'-DFUR would be preferably metabolized to 5-FU in tumor tissue by activity-enhanced thymidine phosphorylase with more than 10 times higher concentration of product compared with other normal tissue [4]. This partial targeted drug delivery was believed to improve the treatment efficacy and tolerance of Cap. It had been reported that approximately 80% of 5-FU is catabolized to inactive product dihydrofluorouracil (FUH2) by the rate-limiting enzyme dihydropyrimidine dehydrogenase (DPD) in liver, and declined activity of DPD often caused a longer retention of 5-FU [5–7]. Finally, FUH2 would be excreted along with urine as *α*-fluoro-*β*-alanine. The antitumor activity of 5-FU usually worked through three metabolic pathways: the first pathway was the anabolism of 5-FU to fluorouridine triphosphate and incorporating the product into RNA, and finally damage the structure of RNA; the second pathway was similar with the first mechanism and deoxyfluorouridine triphosphate was produced to damage the DNA structure; the third way was anabolism of fluorodeoxyuridine monophosphate from fluorouridine monophosphate or 5-fluoro-2'-deoxyuridine (2'-DFUR), and then a ternary complex would be formed with thymidylate synthetase and folic acid to suppress the synthesis of thymidine. Thus, it blocked the *in vivo* synthesis pathway of DNA (Figure 1). Due to a long metabolic pathway and the interindividual genetic variations, the pharmacokinetic parameters of Cap and its metabolites showed significant differences in patients, and therapeutic drug monitoring of Cap and its metabolites was not applied in clinical practice because of its specific regimen, lack of associations between plasma exposure and clinical response and/or toxicity, and the difficulties of simultaneously quantifying Cap and its metabolites. It is difficult for clinicians to precisely evaluate the exposure level of Cap and its metabolites without proper pharmacokinetic parameters. Several LC-MS/MS methods had been developed to solve this problem [8–14], but the relative high requirement of instruments and/or complicated operations and/or long analytical time has delayed their clinical application (Table 1). Thus, the aim of this study was to develop a simple, rapid, and sensitive method for simultaneous determination of Cap and its five metabolites in human plasma and verify its clinical application.

## 2. Materials and Methods

### 2.1. Chemicals and Reagents

All analytes including Cap (Lot: J0713AS), 5'-DFCR (Lot: J1112AS), 5'-DFUR (Lot: J0713A), 2'-DFUR (Lot: J0620A), 5-FU (Lot: A0930AS), fludarabine (Fdb) (Lot: M0501AS) and 5-chlorouracil (5-ClU) (Lot: J1204A) but excepting FUH2 (Lot: 5-PTR-167-1) were purchased from Meilun Biotech Co., Ltd (Dalian City, China). FUH2 was supplied by Toronto Research Chemicals (Toronto, Canada). HPLC-grade acetonitrile was obtained from Merck (Merck Company, Darmstadt, Germany). HPLC-grade formic acid, dimethyl sulfoxide (DMSO), and ammonium acetate were purchased from Tedia Company Inc (Tedia, Fairfield, OH, USA). Ultrapurified water (0.22 *µ*m filter membrane) was self-made in laboratory on a Milli-Q Reagent Water System (Millipore, MA) and was used throughout. HPLC-grade isopropanol and ethyl acetate were bought from Thermo fisher (Thermo Fisher Scientific, Waltham, MA) and Caledon laboratories (Caledon Laboratories Ltd, Georgetown, Canada). Human blank plasma was donated by Shanghai Red Cross Blood Center (Shanghai, China).

### 2.2. LC-MS/MS Instrumentation

All experiments were performed on Agilent 1260 series UHPLC system, which included an online degasser, a binary pump, an autosampler, and column oven and interfaced to an Agilent 6460A triple-quadrupole mass spectrometer equipped with an electrospray ionization source (Agilent Technologies, Santa Clara, CA, USA). All raw data were acquired and analyzed using Agilent Masshunter data processing software (version B.06.00; Agilent Technologies, Santa Clara, CA, USA).

### 2.3. Liquid Chromatographic Conditions

The optimized chromatographic conditions were completed on an Atlantis T3-C_18_ analytical column (3.0 *µ*m, 2.1 × 100 mm; Waters Co, Milford, CT, USA). The column was equilibrated and eluted under gradient phases containing 0.0075% formic acid in water (phase A, pH4) and in acetonitrile (phase B), and the flow rate was 0.3 mL/min. The mobile phases were degassed automatically using the online degasser system. The gradient variation started with 0% phase B. Within 0.5 min, the phase B escalated to 10% and maintained until 2 min and then sharply rose to 90% at 3 min and maintained until 5 min. The total run time was 5 min. The column temperature was maintained at 35°C. The injection volume was 5 *µ*L with a 3-second needle wash using 5% methanol aqueous solution.

### 2.4. Mass Spectrometry Conditions

**Figure 2:**
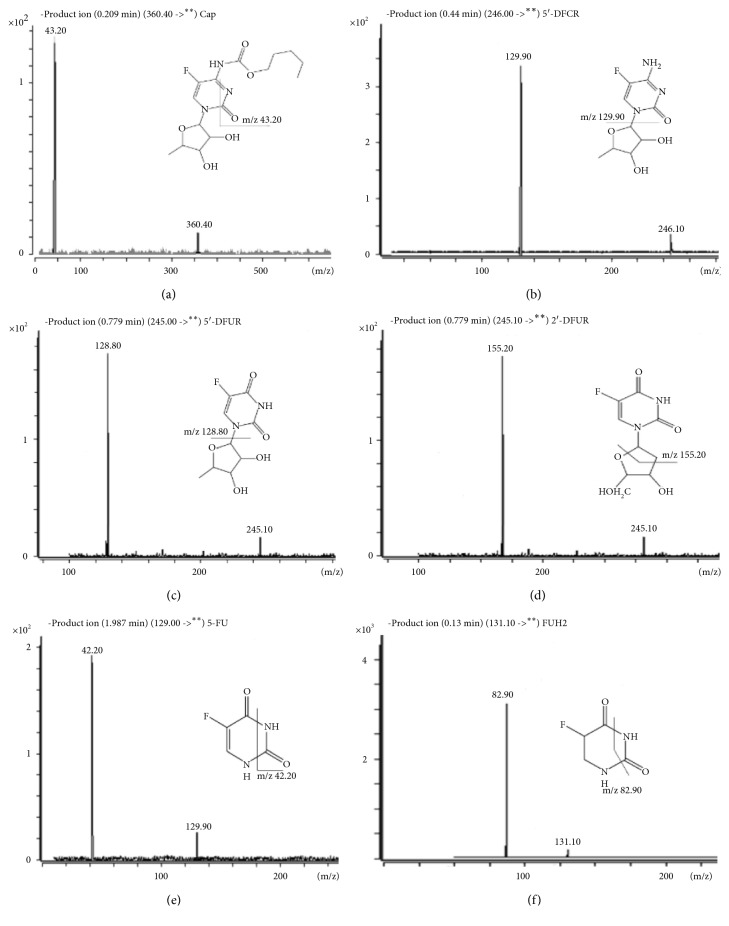
Product ions chromatograms and fragment structures of Cap and its five metabolites. (a) Cap; (b) 5'-DFCR; (c) 5'-DFUR; (d) 2'-DFUR; (e) 5-FU; (f) FUH2.

The mass detection was achieved using electrospray ionization both in the positive and negative modes with the capillary voltage set at 4000 V. Nitrogen was utilized as drying gas, nebulizer gas, and sheath gas. Drying gas was heated to 300°C and delivered at 10 L/min. The temperature of sheath gas was set at 300°C, and the flow rate was 12 L/min. Nebulizer pressure was set at 45 psi. High purity nitrogen served as collision gas at a pressure around 0.2 MPa. Data acquisition was performed in the multiple reaction monitoring (MRM) mode (Figure 2).  Table 2 shows the optimized MRM parameters for Cap and its five metabolites. The peak widths of precursors and product ions were maintained at 0.7 amu at half-height of peak in the MRM mode, and the dwell time for all analytes was 80 ms.

### 2.5. Preparation of Standard and Quality Control Samples

The stock solutions of Cap and its metabolites 5'-DFCR, 5'-DFUR, 2'-DFUR, 5-FU, and FUH2 as well as internal standards (IS) 5-ClU and Fdb were individually prepared in methanol. 2.04, 2.00, 2.04, 2.20, 2.06, and 2.02 mg of Cap, 5'-DFCR, 5'-DFUR, 2'-DFUR, 5-FU, and FUH2 were accurately weighed and dissolved in methanol. Several drops of DMSO were added into the FUH2 solution, and the final volumes of all stock solutions were 2.00 mL. All stock solutions were subpackaged and stored at −80°C. The stock solution of each analyte was further diluted with 10% methanol to obtain a series of work solutions at the following concentrations: 204.0, 510.0, 1020.0, 5100.0, 10200.0, 25500.0, and 51000.0 ng/mL for Cap and 5'-DFUR; 200.0, 500.0, 1000.0, 5000.0, 10000.0, 25000.0, and 50000.0 ng/mL for 5'-DFCR; 220.0, 550.0, 1100.0, 5500.0, 11000.0, 27500.0, and 55000.0 ng/mL for 2'-DFUR; 206.0, 515.0, 1030.0, 5150.0, 10300.0, 25750.0, and 51500.0 ng/mL for 5-FU; and 202.0, 505.0, 1010.0, 5050.0, 10100.0, 25250.0, and 50500.0 ng/mL for FUH2. Calibration standards were prepared by 10 times dilution of the corresponding combined working solutions with blank human plasma to obtain final concentrations in the range of 20.4–5100 ng/mL for Cap and 5'-DFUR; 20.0–5000.0 ng/mL for 5'-DFCR; 22.0–5500.0 ng/mL for 2'-DFUR; 20.6–5150.0 ng/mL for 5-FU; and 20.2–5050.0 ng/mL for FUH2. Quality control (QC) samples were also prepared in the same way (20.4, 51.0, 1020.0, and 2550.0 ng/mL for Cap and 5'-DFUR; 20.0, 50.0, 1000.0, and 2500.0 ng/mL for 5'-DFUR; 22.0, 55.0, 1100.0, and 2750.0 ng/mL for 2'-DFUR; 20.6, 51.5, 1030.0, and 2575.0 ng/mL for 5-FU; 20.2, 50.5, 1010.0, and 2525.0 ng/mL for FUH2). The QC samples were stored at −20°C and brought to room temperature (25°C) for thaw before pretreatment. The IS stock solutions were prepared in the same way. 2.32 mg Fdb and 1.84 mg 5-ClU were individually dissolved in 2 mL methanol, and finally combined IS solution was freshly prepared in the extraction solvent at a concentration of 30 ng/mL for both Fdb and 5-ClU before use. The Fdb was utilized as IS for Cap and 5'-DFCR, 5'-DFUR, 2'-DFUR, and 5-ClU was the IS for 5-FU and FUH2.

### 2.6. Sample Pretreatment

For all analytes, sample pretreatment was performed by a one-step liquid-liquid extraction procedure. A 100 *µ*L aliquot of sample was transferred to a 10 mL glass centrifuge tube prior to adding 3 mL extraction solvent (ethyl acetate : isopropanol = 19 : 1, vol : vol). After 3 min of vortex-mixing, tubes were centrifuged at 1710 × g for 10 min at room temperature. Then, 2.7 mL of organic phase was drawn and transferred to 5 mL plastic centrifuge tubes and evaporated by a SCANVAC Freeze Dryer (Labogene, Shanghai, China) at 1000 × g, 73 bar, and room temperature for 30 min. The residual was reconstituted with 100 *µ*L of 10% methanol aqueous solution, and 5 *µ*L of the reconstituted solution was injected directly to the UHPLC-MS/MS system for analysis.

### 2.7. Human Sample Collection

This research protocol was approved by the Ethical Committee of Changzheng Hospital (Shanghai, China) and performed in the Changzheng Hospital. Informed consent was signed by all recruited patients. Samples were solely utilized to validate this method, and sparse sampling points were applied to collect clinical samples. Venous blood samples were collected in EDTA-3K collecting tubes and gently shaken and then immediately centrifuged at 1710 × g for 10 min. The plasma was collected and stored at −80°C until analysis.

### 2.8. Method Validation

Method validation, including specificity, linearity, inter- and intraprecision and accuracy, matrix effect, recovery, carryover, and stability, was performed according to the Chinese pharmacopeia (version 2015).

For specificity, comparisons of responses in spiked and blank samples from at least six different lots were performed. The responses of interferents not more than 20% of LLOQ sample and 5% of IS were acceptable.

Matrix effect and recovery were assessed in six replicates at two (low and high) concentration levels for all analytes, and 50 and 2500 ng/mL were chosen as the low and high concentration levels. The matrix effect was the ratios of peak area in the spiked postextraction samples to the peak area in solvent-substituted samples in the same concentration, and the recovery was the ratio of peak area in the spiked samples to the peak area in spiked postextraction samples in the same concentration. The IS was assessed for the matrix effect and recovery at 1000 ng/mL.

Inter- and intraprecision and accuracy were assessed in five replicates at four concentration levels (LLOQ, low, middle, and high). Samples were analyzed in three analytical lots in separate days (at least 2 days), and the RSD% for inter- and intraday precision not more than 15% were rational (for LLOQ, not more than 20%). For intra- and interday accuracy, RE% (relative error) within ±15% (for LLOQ, within ±20%) were considered to be acceptable.

Linearity of each analyte was evaluated in three analytical lots in separate days (at least 2 days) along with the precision and accuracy, and at least three calibration curves were assessed in each analytical lot for one analyte. Calibration curves were regressed from IS-adjusted peak area versus corresponding concentrations in at least six calibration standards using a 1/*χ* ^2^ weighted linear least-squares regression model. The LLOQ was the lowest point in the calibration curve. For each concentration point, the deviation of back-calculation in the corresponding calibration curve should be within ±15% (RE%), and the deviation of back-calculation for LLOQ should not go beyond ±20%. Carryover of all analytes was also tested by injecting the highest calibration standard sample prior to injecting a blank sample, and the response of analyte in blank samples not more than 20% of the LLOQ and 5% of the IS was considered to be rational.

Stability, including long-term stability (3 months), short-term stability (24 h in autosampler), and three frozen-thaw cycles stability, was evaluated using QC samples at two concentration levels (low and high). The corresponding calibration curve for each analyte was employed to obtain the measured concentrations, and the deviation from nominal concentration within ±15% (RE%) conformed to the criterion.

## 3. Results and Discussion

### 3.1. Chromatography Condition Optimization

The physicochemical properties of Cap and its metabolites have little in common with each other. Cap had a long carbon chain and showed lipophilicity. After metabolized gradually by the enzyme, the residual structure increased its polarity and showed hydrophilicity. It had reported that only some specific columns could retain simultaneously Cap and its metabolites, for instance, Hypercarb column [10] and Atlantis T_3_ column [11]. However, Hypercarb column needed to cooperate with complex quaternary mobile phases, including water, acetonitrile, 2-propanol, and tetrahydrofuran, and took a long elution time (12 min). Also an early reported method based on Atlantis T_3_ column took a long time (14 min) for analytes separation and did not contain the FUH2. Because of these disadvantages, we developed an optimized method with shorter analytical time (5 min) based on the Altantis T_3_ column and binary mobile phase system. The Altantis T_3_ column, which is optimized for retention polar compounds, can stand 100% water in separation process, and this characteristic was utilized in this method to increase the retention time of 5-FU and FUH2 to 2 min. In addition, some universal columns containing ZORBAX SB-C_18_, Xselect BEH, Xbridge BEH, and Eclipse PLUS-C_18_ were also tested for their retention and separation ability. Unfortunately, enough retention and separation could not be obtained from these universal columns. 0.0075% formic acid in water (pH 4) and in acetonitrile was utilized to elute the analytes and suppress tailing, and optimized retention time and separation were gained after testing different ratio and kinds of acids (formic acid, acetic acid, trifluoroacetic acid, heptafluorobutyric acid; ratio: 0.5%, 0.1%, 0.05%, 0.01%, 0.0075%) in mobile phase. Ammonium acetate as additive in mobile phase could suppress signal response, and methanol decreased the symmetry of peaks.

### 3.2. Sample Pretreatment

Owing to the great difference of physicochemical property between Cap and its metabolites, liquid-liquid extraction by ethyl acetate and isopropanol (19 : 1, vol : vol) was chosen as the extraction solvent which gave optimal extraction recovery and matrix effect in pretreatment procedure. Before that, we tested different proportions and combinations of organic solvents, for example, ethyl acetate, isopropanol, dichloromethane, trichloromethane, methyl tertiary-butyl ether, and cyclohexane. During the pretreatment method development, protein precipitation and solid phase extraction were temporarily utilized as the pretreatment method. For protein precipitation, methanol(1 : 3, vol : vol), acetonitrile(1 : 2, vol : vol), acetone(1 : 2, vol : vol), and 10% trichloroacetic acid(1 : 1, vol : vol) were tested for their ability of deproteinization and removing interferential matrix. The results showed a recovery less than 10% for all the analytes, and then solid phase extraction for all the analytes using Osis HLB, MCX, and MAX cartridge (Waters Co., Milford, CT) were tried. The 5-FU and FUH2 could not be retained in these cartridges, and ion-exchange solid phase extraction was also utilized based on the Plexa PCX cartridge (Agilent Technologies, Santa Clara, CA) to extract all analytes according to the instructions. But this might be restricted by the chemical structure of Cap and its metabolites. There were several nitrogen atoms in both Cap and its metabolites, which would carry out ion-exchange process in given chemical circumstance, but the ringlike structure and steric hindrance might hinder the formation of ammonium and succedent ion-exchange. Finally, the Cap and its metabolites could not be retained adequately in the ion-exchange cartridge.

### 3.3. Method Validation

#### 3.3.1. Specificity

**Figure 3:**
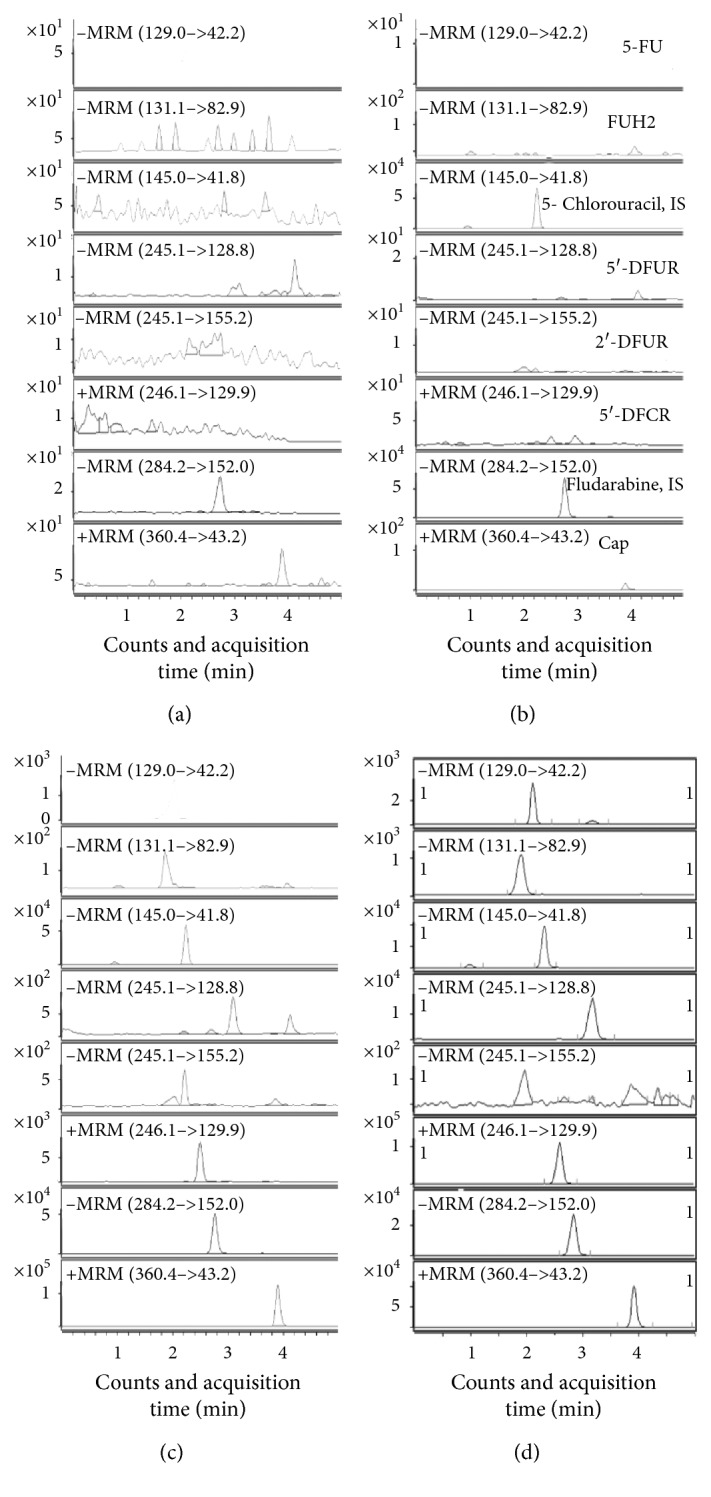
Representative MRM chromatograms of Cap and its five metabolites. (a) Blank sample; (b) blank sample spiked IS; (c) blank sample spiked with LLOQ concentration of Cap and its metabolites; (d) real sample collected from one colorectal cancer patient after administration of Cap.

Comparisons of chromatograms from blank, IS spiked, LLOQ, and real samples (Figure 3) indicated that there were not any significant interferences at the same retention times of the analytes and IS.

#### 3.3.2. Linearity of Calibration Curves and LLOQ

Calibration curves were constructed by calculating the peak area ratios (analyte/IS) of calibration standards versus measured concentrations. Seven calibration standards were obtained from spiked samples, and the best linearity and least-squares residuals for the calibration curves were achieved with a 1/χ^2^ weighing factor. The linear correlation coefficients were more than 0.99 for all analytes. Typical regression equations for the calibration curves are summarized in Table 3. The LLOQs were all around 20 ng/mL in human plasma matrix, which were also in accordance with the accuracy within ±20% and precision less than 20%. These LLOQs were more sensitive than some previously reported method and sufficient for monitoring of Cap and its metabolites in clinical practice.

**Figure 4:**
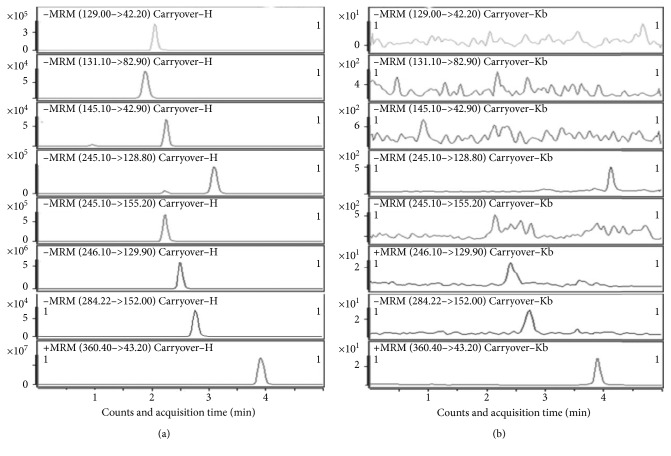
Comparative chromatograms of carryover of Cap and its five metabolites. (a) Highest calibration standard sample and (b) blank sample.

Carryover between samples often caused confusing results to the lower ones. In this method, three cycles of highest-blank samples were injected orderly to assess responses in the blank samples. The results showed that responses in blank sample were less than 20% of the LLOQ and 5% of the IS (Figure 4).

#### 3.3.3. Inter- and Intraprecision and Accuracy

Four levels of QC samples (LLOQ, low, middle, and high) were chosen to analyze the inter- and intraprecision and accuracy. The results showed a good precision and accuracy with intra- and interprecision less than 10.45% and accuracy within ±15% (LLOQ within ±20%).  Table 4 summarizes the inter- and intraday precision and accuracy for the six analytes.

#### 3.3.4. Matrix Effect and Recovery

The liquid-liquid extraction commonly could remove the endogenous interferents at the greatest extent, while the protein precipitation left the most serious matrix interference [15]. Researchers had reported the severe ion suppression for the downstream products of Cap, such as FUH2 and 5-FU, using the protein precipitation as sample pretreatment method [12]. Severe ion suppression often caused by the coeluted interferents, for example, lipids and some polar small molecular compounds, and liquid-liquid extraction with ethyl acetate and isopropanol in this method could eliminate more polar small molecular compounds. So, the results showed an immensely declined matrix effect which ranged from 74.84% to 114.48% compared with other reported methods, and the recovery ranged from 59.27% to 90.15%. The IS-normalized matrix and recovery factor were calculated following the acquisition of matrix effect and recovery, and the results showed that the RSD (%) of matrix and recovery factors was not more than 15% (Table 5). The matrix effect and recovery were stable and conformed to the criterion for all the analytes and IS.

#### 3.3.5. Stability

The stability of analytes containing long-term stability, short-term stability, and three frozen-thaw cycles was investigated at two concentration levels (low and high). The analytes were found to be stable in human plasma for 3 months at −80°C and in autosampler at 4°C for 24 h (<10% reduction). After three freeze-thaw cycles, no obvious deviations (within ±15%) were observed for all analytes (Table 5).

### 3.4. Application in Determination of Clinical Samples

Totally, 42 sparse samples were collected from 36 colorectal cancer patients who were treated with 1000 mg/m^2^ Xeloda tablet at the Changzheng Hospital. Thirty-one samples were collected on 31 patients, and the time points ranged from 0.5 to 9 h after administration. Nine samples were harvested from 3 patients at 1, 2.5, and 4 h and the other 2 samples originated from 1 patient at 1 and 4 h after administration. The analytes were quantitatively measured by this fully validated UHPLC-MS/MS method in the plasma. As a result, apparent differences of drug concentrations in plasma were found in Cap and its metabolites in absorption and metabolism processes, and several studies also reported these differences [16–20]. The drug exposure *in vivo* has a close relationship with the treatment efficacy and/or side effects, and it was still the promising biomarker for the clinical prognosis. The AUC of 5-FU was reported to associate with the myelosuppression and mucositis as well as the treatment response [21]. A timely reflection of the drug exposure might be vital for a better treatment outcome and alleviation of side effects. As the samples were collected in sparse points, no definite pharmacokinetic parameters were gained in this validation process (Supplementary Table S1).

## 4. Conclusion

A simple, rapid, and sensitive UHPLC-MS/MS method was successfully developed and validated, which was suitable for simultaneous determination of Cap and its five metabolites in human plasma from colorectal cancer patients. The LLOQ was approximately 20 ng/mL, and the analytical time was 5 min for all analytes after optimizing the chromatography separation and mass spectrometer detection conditions. With a simple sample pretreatment, this method was suitable for clinical therapeutic drug monitoring of Cap and its metabolites to get a better treatment outcome.

## Figures and Tables

**Table 1:** Comparative summary of developed LC-MS/MS methods for quantification of Cap and its five metabolites.

Author	Instrument	Pretreatment method	Analytical time (min)	Range (ng/mL)	Number of quantified analytes
Montange et al. [8]	LC-MS/MS	Liquid-liquid extraction	15	150–3000 (Cap)	4
				400–8000 (5'-DFUR)	
				200–4000 (5'-DFCR)	
				50–1000 (5-FU)	

Licea-Perez et al. [9]	LC-MS/MS	Liquid-liquid extraction and derivatization	4.5 + 2.5(2 independent analytical processes)	10–10000 (FBAL)	3
				5–5000 (5-FU)	
				1–1000 (Cap)	

Vainchtein et al. [10]	LC-MS/MS	Protein precipitation	12	10–5000 (5'-DFCR, 5'-DFUR)	5
				10–1000 (Cap)	
				50–5000 (5-FU, FUH2)	

Salvador et al. [11]	LC-MS/MS	Solid-phase extraction	14	1–500 (Cap, 5-FU)	4
				10–1000 (5'-DFCR, 5'-DFUR)	

Deenen et al. [12]	LC-MS/MS	Two independent protein precipitation procedures	9 + 5(2 independent analytical procedures)	50–6000 (Cap, 5'-DFCR, 5'-DFUR)	7
				50–5000 (5-FU, FUH2, FUPA, FBAL)	

Siethoff et al. [13]	LC-MS/MS	Protein precipitation and derivatization	8	5–1000 (Cap, 5-FU)	2

Wang et al. [14] ^a^(new method)	LC-MS/MS	One-step liquid-liquid extraction	5	20–5000 (Cap, 5'-DFCR, 5'-DFUR, 2'-DFUR, 5-FU, FUH2)	6

LC-MS/MS, liquid chromatography tandem mass spectrometry; Cap, capecitabine; 5'-DFCR, 5'-deoxy-5-fluorocytidine; 5'-DFUR, doxifluridine; 2'-DFUR, 5-fluoro-2'-deoxyuridine; 5-FU, 5-fluorouracil; FUH2, dihydrofluorouracil; FUPA, *α*-fluoro-*β*-ureidopropionic acid; FBAL, *α*-fluoro-*β*-alanine. ^a^The newly developed method described in this article.

**Table 2:** Optimized mass spectrometry parameters of Cap and its five metabolites.

Analytes	Ionization mode	Precursor ions (m/z)	Fragmentor (V)	Collision energy (V)	Product ions (*m*/*z*)
Cap	+	360.4	70	5	43.2
5'-DFCR	+	246.1	110	5	129.9
5'-DFUR	−	245.10	75	11	128.8
2'-DFUR	−	245.1	75	9	155.2
5-FU	−	129.00	90	17	42.2
5-CLU (IS)	−	145.00	110	18	41.8
FUH2	−	131.1	75	5	82.9
Fdb (IS)	−	284.22	125	13	152

**Table 3:** Linearity regression parameters of Cap and its five metabolites.

Analytes	Regression type	Regression equations	LLOQ (ng/mL)	Linear range	Weight	*r*

Cap	Quadratic	*Y* = −157.20 ∗ *x* ^2^ + 198.88 ∗ *x* + 0.2^a^	20.40	20.4–5100.0	1/*x* ^2^	0.995
5'-DFCR	Linearity	*Y* = 77.69 ∗ *x* − 0.03	20.00	20.0–5000.0	1/*x* ^2^	0.997
5'-DFUR	Linearity	*Y* = 6.57 ∗ *x* + 1.08	20.40	20.4–5100.0	1/*x* ^2^	0.997
2'-DFUR	Linearity	*Y* = 4.06 ∗ *x* + 5.82	22.00	20.6–5150.0	1/*x* ^2^	0.995
5-FU	Linearity	*Y* = 9.08 ∗ *x* + 0.02	20.60	20.6–5150.0	1/*x* ^2^	0.993
FUH2	Linearity	*Y* = 1.55 ∗ *x* − 4.98	20.20	20.2–5050.0	1/*x* ^2^	0.997

^a^Quadratic was selected as the upper limit of response approached by Cap in mass spectrometry detection.

**Table 4:** Inter- and intraprecision and accuracy of Cap and its 5 metabolites (*n* = 5).

Analyte	Nominal Concentration (ng/mL)	Intraday			Interday		
		Measured concentration(ng/mL, mean ± SD)	Precision (RSD%)	Accuracy (RE%)	Measured concentration (ng/mL, mean ± SD)	Precision (RSD%)	Accuracy (RE%)
Cap	20.40	19.70 ± 1.54	8.24	−3.40	19.30 ± 1.58	8.04	−5.70
	51.00	48.61 ± 4.91	10.09	−4.79	50.07 ± 2.78	5.20	−2.15
	1020.00	924.82 ± 58.80	6.36	−9.33	991.58 ± 86.25	8.33	−3.13
	2550.00	2535 ± 37.93	1.50	−0.56	2306.09 ± 160.75	6.69	−9.88

5'-DFCR	20.00	22.82 ± 0.44	1.94	14.13	21.78 ± 1.01	4.65	8.88
	50.00	46.77 ± 0.62	1.33	−6.46	46.92 ± 1.39	2.97	−6.15
	1000.00	977.35 ± 17.15	1.75	−2.27	1014.99 ± 48.90	4.82	1.50
	2500.00	2614.20 ± 23.70	0.91	4.57	2553.80 ± 74.24	2.91	2.15

5'-DFUR	20.40	23.52 ± 0.88	3.75	15.29	22.74 ± 1.25	6.16	8.53
	51.00	53.51 ± 1.69	3.16	4.91	52.35 ± 2.13	4.53	0.11
	1020.00	1011.02 ± 20.72	2.05	−0.89	1108.28 ± 7.05	4.83	5.82
	2550.00	2625.62 ± 25.97	0.99	2.97	2642.50 ± 154.56	3.06	0.94

2'-DFUR	22.00	23.20 ± 2.36	10.19	5.45	21.93 ± 2.37	6.27	0.59
	55.00	55.53 ± 2.16	3.89	0.96	52.80 ± 3.83	4.45	−1.11
	1100.00	1100.18 ± 36.89	3.35	0.02	1134.77 ± 57.84	4.48	5.77
	2750.00	2741.87 ± 46.13	1.68	−0.29	2635.74 ± 88.38	2.69	−1.73

5-FU	20.60	19.94 ± 1.35	6.77	−3.31	19.50 ± 1.62	8.30	−5.52
	51.50	52.91 ± 1.21	2.28	2.73	52.91 ± 2.55	4.81	2.73
	1030.00	1082.02 ± 22.35	2.06	5.05	1094.09 ± 22.64	2.07	6.22
	2575.00	2443.83 ± 14.00	0.57	−5.10	2432.66 ± 28.22	1.16	−5.53

FUH2	20.20	22.90 ± 1.45	6.34	13.39	21.15 ± 2.21	10.45	4.69
	50.50	48.46 ± 4.09	8.43	−4.05	47.93 ± 3.35	6.99	−5.10
	1010.00	936.66 ± 23.46	2.50	−7.26	961.43 ± 38.60	4.01	−4.81
	2525.00	2521.59 ± 2.68	2.68	−0.13	2579.34 ± 106.56	4.13	2.15

**Table 5:** Recovery, matrix effect, and stability of Cap and its five metabolites (%).

		Cap	5'-DFCR	5'-DFUR	2'-DFUR	5-FU	FUH2	5-ClU	Fdb
Frozen-thaw stability	Low	93.25	95.23	98.35	100.57	97.35	93.34	—	—
	High	95.12	93.13	96.56	86.87	87.23	99.31	—	—
24 h in autosampler	Low	99.52	101.91	113.92	110.25	109.94	100.33	—	—
	High	94.62	114.62	114.04	112.53	100.04	108.73	—	—
Long-term stability (3 months)	Low	95.23	96.32	106.15	102.53	106.78	101.45	—	—
	High	98.55	95.65	110.25	112.25	96.25	105.22	—	—
Recovery	Low	90.15	80.89	70.26	59.27	64.39	88.50	87.30	91.64
	High	85.26	80.89	70.26	59.86	76.82	82.12		
	RSD^*∗*^	3.69	3.04	4.51	4.51	10.39	3.42	—	—
Matrix effect	Low	114.48	110.24	106.62	106.93	74.84	94.14	106.10	60.91
	High	108.94	109.08	106.31	105.63	83.55	93.00		
	RSD^*∗*^	3.84	2.70	4.39	3.87	5.69	4.78	—	—

^*∗*^RSD was calculated using the IS-normalized matrix and recovery factors.

## Data Availability

The data used to support the findings of this study are available from the corresponding author upon request.

## Abbreviations

Cap: Capecitabine,
5'-DFCR: 5'-Deoxy-5-fluorocytidine
5'-DFUR: Doxifluridine
2'-DFUR: 5-Fluoro-2'-deoxyuridine
5-FU: 5-Fluorouracil
FUH2: Dihydrofluorouracil
IS: Internal standard
Fdb: Fludarabine
5-ClU: 5-Chlorouracil
DPD: Dihydropyrimidine dehydrogenase
UHPLC-MS/MS: Ultrahigh-performance liquid chromatography tandem mass spectrometry
DMSO: Dimethyl sulfoxide
MRM: Multiple reaction monitoring
LLOQ: Lower limit of quantification.

## Supplementary Materials

Supplementary Materials Totally, 42 sparse samples were utilized to validate this newly developed method. The results showed a satisfying linear range, which covered Cap and its metabolites exposure in vivo in different sampling points, and interpatient variation of exposures was displayed when compared samples collected at the same time from different patients. This method may pave the road for clinically monitoring of Cap and its metabolites and therapeutic regimen optimization.
