# Supplementary material for: A Sensitive and Efficient Method for Determination of Capecitabine and Its Five Metabolites in Human Plasma Based on One-Step Liquid-Liquid Extraction
Source: J Anal Methods Chem. 2019 Jan 3;2019:9371790. doi: 10.1155/2019/9371790 (PMC6335671; doi:10.1155/2019/9371790)
Supplement: Supplementary Materials — Totally, 42 sparse samples were utilized to validate this newly developed method. The results showed a satisfying linear range, which covered Cap and its metabolites exposure in vivo in different sampling points, and interpatient variation of exposures was displayed when compared samples collected at the same time from different patients. This method may pave the road for clinically monitoring of Cap and its metabolites and therapeutic regimen optimization. [file 9371790.f1.docx]

| No. | t (min) | Cap | 5’-DFCR | 5’-DFUR | 2’-DFUR | 5-FU | FUH2 |
| --- | --- | --- | --- | --- | --- | --- | --- |
| 1 | 120 | 200.74 | 4161.63 | 2417.67 | - | 84.69 | 744.12 |
| 2 | 290 | 120.00 | 53.95 | 451.77 | 45.86 | 31.32 | 96.43 |
| 3 | 230 | 536.75 | 1216.60 | 755.09 | - | - | 329.00 |
| 4 | 41 | 4902.81 | 4795.83 | 3517.86 | - | 222.85 | 345.60 |
| 5 | 240 | 2063.20 | 3743.84 | 2514.97 | - | 72.39 | 772.52 |
| 6 | 185 | 3830.71 | 5138.52 | 3756.23 | - | 117.82 | 694.32 |
| 7 | 41 | 4342.05 | 911.91 | 398.50 | - | 22.74 | 48.06 |
| 8 | 270 | 497.87 | 332.41 | 190.52 | - | - | 55.82 |
| 9 | 80 | 1077.11 | 511.41 | 335.13 | - | 67.89 | - |
| 10 | 540 | - | - | 30.30 | - | 30.89 | 22.96 |
| 11 | 175 | 1317.75 | 2723.70 | 1537.28 | - | 64.57 | 299.15 |
| 12 | 100 | 1739.45 | 4460.34 | 3330.95 | 34.89 | 95.01 | 792.98 |
| 13 | 125 | 1385.84 | 1451.58 | 1163.68 | - | 38.81 | 181.02 |
| 14 | 270 | 599.46 | 2319.19 | 1499.88 | - | 37.81 | 538.71 |
| 15 | 30 | 311.86 | 224.55 | 169.54 | - | - | - |
| 16 | 305 | 563.27 | 1411.67 | 791.82 | - | 25.28 | 317.79 |
| 17 | 180 | 851.14 | 890.05 | 685.76 | - | - | 87.87 |
| 18 | 52 | 3505.71 | 750.39 | 535.52 | - | - | 38.40 |
| 19 | 40 | 1609.76 | 825.74 | 515.62 | - | - | 38.28 |
| 20 | 380 | - | 146.15 | 64.92 | - | - | 218.06 |
| 21 | 180 | 95.03 | 827.91 | 724.97 | - | 22.55 | 441.21 |
| 22 | 385 | 74.93 | 2820.74 | 113.22 | - | - | 128.24 |
| 23 | 60 | 262.85 | 134.56 | 41.13 | 27.29 | 85.59 | - |
| 24 | 220 | 411.45 | 270.03 | 134.68 | - | - | 46.26 |
| 25 | 330 | - | 31.02 | - | - | - | 41.81 |
| 26 | 150 | 4792.45 | 4270.40 | 3430.85 | 80.89 | 91.75 | 514.38 |
| 27 | 325 | 610.93 | 1561.04 | 1283.00 | - | 29.64 | 430.07 |
| 28 | 150 | 165.78 | 164.61 | 68.40 | 74.30 | 198.13 | 49.16 |
| 29 | 175 | 1454.98 | 3634.65 | 2441.92 | - | 107.92 | 1006.93 |
| 30 | 30 | 113.10 | 485.94 | 462.32 | - | 18.91 | 243.44 |
| 31 | 140 | 305.96 | 159.93 | 142.43 | - | - | - |
| 32 | 60 | 252.04 | 172.55 | 259.76 | - | - | 43.30 |
| 33 | 150 | 835.97 | 1658.13 | 2280.61 | - | - | 623.02 |
| 34 | 240 | 110.94 | 320.34 | 498.91 | - | - | 258.58 |
| 35 | 60 | 275.12 | 165.92 | 102.60 | - | - | - |
| 36 | 240 | 1512.73 | 2401.12 | 1199.89 | - | 20.25 | 494.55 |
| 37 | 60 | 51.62 | 36.50 | - | - | - | - |
| 38 | 150 | 475.16 | 899.99 | 699.78 | - | - | 143.30 |
| 39 | 240 | 536.92 | 1181.43 | 1083.58 | - | 20.46 | 279.09 |
| 40 | 60 | 25.44 | - | - | - | - | - |
| 41 | 150 | 2219.90 | 1077.45 | 2243.60 | - | - | 160.87 |
| 42 | 240 | 439.69 | 373.04 | 688.25 | - | - | 164.95 |

Table s1. Plasma exposures of Cap and its metabolites in colorectal cancer patients quantifying by new developed method.

Unit: ng/mL; t: sampling time after administration of Capecitabine tablet. No.: sample number.
